# Prevalence of Mental Disorders in Parents With Intellectual Disabilities: A Systematic Review

**DOI:** 10.1111/jir.70167

**Published:** 2026-08-27

**Authors:** Sonya Rudra, Louise Marston, Angela Hassiotis, Giovanni Boido, Mary Baginsky, Claire A. Wilson

**Affiliations:** ^1^ Division of Psychiatry University College London London UK; ^2^ Department of Primary Care and Population Health University College London London UK; ^3^ Department of Health Studies University of Milan Milan Italy; ^4^ Department of Mental Health and Addiction ASST Santi Paolo e Carlo Milan Italy; ^5^ NIHR Health and Social Care Workforce Research Unit, Policy Institute King's College London London UK; ^6^ South London and Maudsley NHS Foundation Trust London UK; ^7^ Institute of Psychiatry, Psychology and Neuroscience Kings College London London UK

**Keywords:** intellectual disabilities, mental disorders, parents, perinatal, prevalence

## Abstract

**Background:**

Research indicates that an increasing number of adults with intellectual disabilities (IDs) are becoming parents. Parents with ID face multiple challenges that increase their mental health risks. In order to ensure better detection and tailored care for parents with ID, this systematic review aimed to summarise the prevalence of mental disorders in parents with ID during pregnancy, postpartum and parenthood.

**Methods:**

Studies included individuals with clinically diagnosed ID who had experienced pregnancy or parenthood and reported prevalence figures of mental disorders. Searches of MEDLINE, APA PsycINFO, EMBASE and grey literature were conducted from inception to July 2025. Risk of bias was assessed using the Mixed Methods Appraisal Tool.

**Results:**

The review identified 10 studies involving 42 207 individuals aged 18–58 years. Across the studies, the prevalence of mental disorders specifically during pregnancy was not reported; two studies screened women within 12 months postpartum; the remaining studies included parents of children aged 0–21 years. The results indicate an overall prevalence of 45% for any mental disorder in parents with ID. Studies using screening tools reported prevalence rates of up to 63% for depression, 30% for anxiety and 17% for psychosis. One large cohort study assessing mental health over the perinatal period reported increased odds of all mental disorders in mothers with ID. Due to the heterogeneity of methodologies used across studies data was not pooled.

**Conclusion:**

This review indicates a high prevalence of mental disorders in parents with ID. The findings highlight the need for tailored screening and support for parents with ID. However, literature is limited and more research is required.

## 1 Introduction

Parenthood is a major life transition that can confer both psychological benefits and substantial emotional, social and physical demands. Across the general population, the perinatal period, defined here as pregnancy and the first year following childbirth, and the early years of parenthood, represent times of heightened vulnerability to mental disorders. In particular, depression, anxiety, bipolar disorder and postpartum psychosis contribute to maternal morbidity and adversely affect infant development and family functioning (Howard et al. 2014; O'Hara and Wisner 2014). Beyond the perinatal period, parenting‐related stress has consistently been associated with an increased risk of depression and anxiety (Al‐Thiab et al. 2026), suggesting that the demands of parenting may contribute to the onset or exacerbation of mental disorders.

Although extensive evidence exists regarding parental mental health in the general population, less attention has been paid to parents with intellectual disability (ID). Historically, people with ID were often denied opportunities to establish intimate relationships and become parents because of institutionalisation, restrictive policies and assumptions regarding parenting capacity. However, deinstitutionalisation, changing legislation, increasing recognition of reproductive rights and improved community support have resulted in growing numbers of people with ID becoming parents (Turney et al. 2018). Consequently, understanding the health needs of this population has become an increasingly important clinical and public health issue.

There are compelling reasons to hypothesise that parents with ID may be at increased risk of mental disorders. Adults with ID experience higher rates of mental disorders than the general population (Deb et al. 2001; Cooper et al. 2007). Parenthood may introduce additional stressors, including the demands of caring for children while managing cognitive impairments, increased surveillance by health and social care professionals and disproportionate involvement with child protection services (Booth and Booth 1994). Mothers with ID are more likely than mothers without ID to experience socioeconomic disadvantage (Mitra et al. 2015; Fairthorne et al. 2020; Tarasoff et al. 2020). They also frequently report unmet support needs, including limited emotional and practical support and difficulties accessing appropriate services (Koolen et al. 2019). Fathers with ID remain comparatively under‐researched and may experience additional barriers arising from their reduced visibility within maternity, parenting and mental health services (Symonds et al. 2021). These overlapping biological, psychological and social vulnerabilities suggest that parents with ID may be particularly susceptible to mental disorders.

Existing research provides only a partial understanding of this issue. Studies have reported elevated rates of mental disorders among parents with intellectual and developmental disabilities (IDDs) (Brown et al. 2016; Brown et al. 2022). However, IDD encompasses a heterogeneous group of lifelong neurodevelopmental conditions, including autism spectrum disorder, cerebral palsy and other developmental disabilities that may occur with or without co‐occurring ID. Combining these populations limits understanding of the specific mental health needs of parents with ID and may obscure important differences in prevalence, aetiology and service needs. A systematic review of pregnancy and postnatal outcomes focused only on women with ID identified 24 obstetric outcomes in nine studies, none of which were mental health related (Lo et al. 2025).

Given the increasing number of parents with ID, the well‐established importance of parental mental health for both parents and children and the unique social and clinical challenges experienced by this population, robust estimates of the prevalence of mental disorders are essential. Such evidence would inform clinical awareness, service planning, early identification and targeted interventions while helping to address longstanding inequities in mental healthcare experienced by people with ID. To date, however, the evidence has not been comprehensively synthesised.

Therefore, the primary aim of this systematic review was to synthesise the evidence on the prevalence of mental disorders among parents with ID during pregnancy, the postpartum period and later parenthood. A secondary aim was to compare the prevalence of mental disorders reported in parents with ID with that reported in non‐parents with ID or parents without ID, where such comparisons were available.

## 2 Methods

This systematic review followed Preferred Reporting Items for Systematic Reviews and Meta‐Analyses (PRISMA) 2020 guidance (Page et al. 2021) and registered with International Prospective Register of Systematic Reviews (PROSPERO; Page et al. 2018). The review included studies of individuals of any age with clinically defined ID defined according to recognised diagnostic classification systems in use at the time of the study (e.g., DSM‐IV, DSM‐5, ICD‐10 or ICD‐11), or common genetic syndromes (e.g., Down syndrome and fragile X syndrome) associated with near 100% penetrance of ID. These syndromes were included because affected individuals may present with mild ID, and individuals with milder ID are more likely to become parents. Eligible studies included birthing people, mothers, fathers or partners. The primary outcome was the prevalence of clinically diagnosed, self‐reported or screened mental disorders during pregnancy, postpartum or parenthood. Where studies included results for a mixed population (e.g., IDD), at least 50% of the sample had to meet criteria for ID or separate results given for those with ID for the study to be included. There were no restrictions by study date, country or language. We excluded self‐report ID, algorithmic/composite ID definitions, borderline intellectual functioning and cohorts with a potentially increased risk of mental disorder (e.g., those with intimate partner violence or involved in child protection proceedings).

The search included all quantitative studies, including case–control, cross‐sectional and cohort designs. Qualitative studies were included if they reported a quantitative component. Intervention studies were included if they provided baseline data about mental disorders. Pre‐prints and articles from non‐peer‐reviewed sources were included and decisions made about whether to include them following the risk of bias assessment stages.

### 2.1 Search Strategy

The search strategy was developed with a specialist librarian, peer‐reviewed (PRESS) (McGowan et al. 2016) and validated against studies identified in a preliminary scoping review. The search strategy combined three core concepts using the Boolean operator AND, with all terms within each concept linked using OR.

The first concept focused on intellectual disability and included subject headings: *Intellectual Disability*, *Fragile X Syndrome*, and *Down Syndrome*, alongside free‐text searches including: intellectual* disab* (or disorder* or difficult*), learning disabilit*, learning difficult*, mental* retard*, mental* handicap*, developmental disabilit*, fragile x, martin‐bell, FXS, Fraxa, marker x syndrome, down* syndrome, and trisomy 21*.

The second concept addressed parents and the perinatal period and included subject headings: *Parents*, *Pregnancy*, *Postpartum Period*, *Perinatal Care*, and *Postnatal Care*, alongside free‐text searches for: parent*, mother*, father*, pregnan*, prenatal, antenatal, perinatal, postnatal, postpartum, and variant spellings (*pre‐natal, ante‐natal, peri‐natal, post‐natal, post‐partum*).

The third concept captured mental disorders using the *Mental Disorders* subject heading and extensive free‐text terms relating to mental disorders: psychiatri*, hypochondriasis, conversion disorder*, obsessive‐compulsive disorder, OCD, adjustment disorder*, PTSD, post‐traumatic stress disorder, eating and feeding disorder*, binge eat*, anorex*, bulimi*, delusion*, bipolar disorder, BPAD, mania, cyclothymi*, psychosis, psychotic disorder*, schizophrenia‐spectrum terms, catatonia, somatic disorder*, depression, depressive disorder*, dysthymi*, anxiety, phobia*, panic disorder*, personality disorder*, paraphilic disorder*, impulse control disorder*, mental condition* (or disorder*, ill*, diagnosis, problem*, health*), affective disorder* (or problem*), mood disorder* (or problem*), compulsive eat* (or vomit* or purg*), substance dependenc* (or addiction, abuse, misuse, disorder*), and dissociative disorder*.

The full search strategy is available in Supporting Information S1: Section S1.

### 2.2 Literature Searching and Data Collection

Searches were conducted on MEDLINE, APA PsycINFO and EMBASE via OVID from inception to April 2025, plus grey literature (PsycEXTRA, PsyArXiv, ProQuest Dissertations and Theses). Searches were also conducted on reference lists from key reviews and to identify corresponding papers from relevant conference abstracts. Duplicates were removed using the Falconer (2018) method, imported into Rayyan for screening and citation tracking completed in Scopus and Web of Science. Database search alerts were set in order to identify any new publications prior to starting the data extraction in July 2025.

Reviewers independently screened titles/abstracts (20% double‐screened with full agreement) and non‐English abstracts were translated. Both reviewers assessed all full texts against eligibility criteria. Initial agreement between the two reviewers was 77% (Cohen's *κ* = 0.44). Following discussion, consensus was reached for 94% (Cohen's *κ* = 0.80) of studies, with the remaining three studies referred to a third reviewer for arbitration. For eligible studies, the reviewers independently extracted study characteristics, ID definitions, outcomes and comparator details using a piloted form; authors were contacted for missing data if required.

### 2.3 Risk of Bias Assessment

The Mixed Methods Appraisal Tool (MMAT; Hong et al. 2018) was used to assess the risk of bias within studies. This tool was chosen for its ability to evaluate qualitative, quantitative and mixed methods studies, as all these study designs were eligible for inclusion in the review. The MMAT uses two universal screening questions and five design‐specific criteria, rated as ‘Yes’, ‘No’ or ‘Cannot tell’. The studies were rated by two reviewers independently. To aid comparisons across studies, a final judgement was given by each reviewer depending on whether the study met the criteria on all five questions (low risk of bias), four questions (moderate risk of bias) or less than four questions (high risk of bias). Any missing or unclear data was judged to increase the overall risk of bias of the paper. Ratings were combined and any disagreements resolved through discussion between the two reviewers.

### 2.4 Data Synthesis

Results are presented in a narrative analysis, based on study characteristics, prevalence estimates and, where available, comparisons with non‐parents with ID or parents without ID. This approach enabled findings from studies with diverse populations, diagnostic definitions and outcome measures to be synthesised while preserving important methodological differences between studies. Meta‐analyses were not deemed appropriate due to the heterogeneity in the definitions of ID, ascertainment of mental disorders, study populations and methodological approaches across the included studies.

## 3 Results

The search yielded 12 957 records from databases and 19 papers via other methods (grey literature, citation searching and papers related to conference abstracts). After deduplication 8322 were screened; 47 full texts assessed; and 10 studies met inclusion criteria. See Supporting Information S1: Section S2 for articles excluded. The study selection process is illustrated in Figure 1.

**FIGURE 1:**
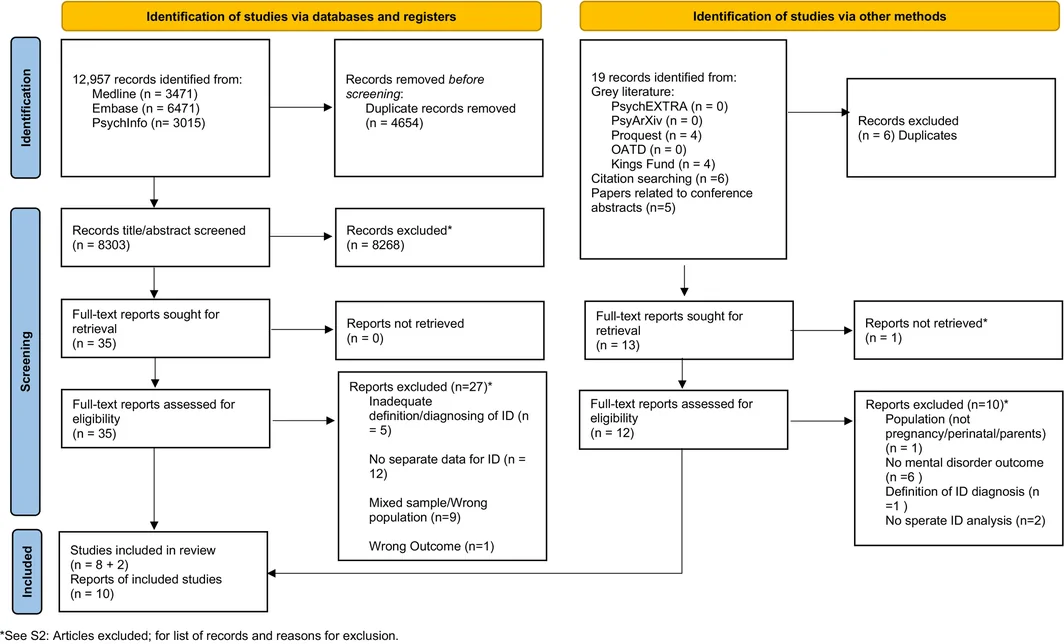
PRISMA flow diagram.

### 3.1 Study Characteristics

The search identified a combination of cross‐sectional studies (Sterling 1998; Tymchuk 1994; Walton‐Allen 1993; Tymchuk 1993; Gaskin and James 2006; McGaw et al. 2010, 2007), cohort studies (Shea et al. 2024; Lindblad et al. 2024) and a qualitative study with quantitative component (Heifetz et al. 2019). Due to the dearth of literature available, two thesis papers from grey literature searches were included in the analysis (Walton‐Allen 1993; Sterling 1998). All studies identified were from high‐income countries: United States (Tymchuk 1994; Shea et al. 2024; Sterling 1998; Tymchuk 1993), United Kingdom (McGaw et al. 2007, 2010; Gaskin and James 2006), Sweden (Lindblad et al. 2024) and Canada (Heifetz et al. 2019; Walton‐Allen 1993). Table 1 shows the characteristics of the included studies.

**TABLE 1:** Study characteristics. Summary of data from the studies included in the review.

Author, year	Country of study sample	Study design	Exposed cohort sample size	Exposed cohort source	Exposed cohort characteristics	Definition/assessment of ID	Outcome (prevalence)	Method to ascertain mental disorder diagnosis	Timing of diagnosis (e.g., age of child/stage of pregnancy/years postpartum)
Sterling 1998	United States	Cross‐sectional	74	Clients of Tri‐Counties Regional Centre and Pomona‐San Gabriel Regional Center, recruited via case manager referral	**Sex**: All females **IQ level** (using Stanford‐Binet Intelligence Scale: Fourth Edition): M = 53.25, SD = 14.5 **Age (years)**: M = 33, R = 18–49 **Ethnicity**: Hispanic = 39.3%, Caucasian = 39.3%, African‐American = 10.7%, Asian = 6.7%, Other = 4% **Marital status**: Married = 38.9%, Single = 43.1%, Living with partner (not married) = 6.9%, Divorced = 8.3%, Widowed = 2.8% **Living situation**: With spouse/partner = 41.1%, Alone with children = 20.8%, Extended family = 27.8%, Group home with staff support = 9.7% **Income**: Below poverty line (< $12 000/year) = 77.5% **Number of children**: one child = 43.7%, two children = 25.3%, three children = 18.7%, four children = 6.7%, five children = 4%, eight children = 1.3% **Language**: Non‐English speaking = 10.8%	IQ ≤ 70 with onset before age 18y and concurrent deficits in adaptive functioning (identified by Regional Centre)	**Depression**: M = 17.64 (SD = 7.68) 77.3% scored above 12/40 (cutoff for assessment of clinical depression) 56% scored above 16/40 (possible clinical depression)	Centre for Epidemiologic Studies Depression Scale	Children's age: R = 4 months to 10 years
McGaw et al. 2007	United Kingdom	Cross‐sectional	49 (only extract data for 30 males)	Families referred to a parenting service specialising in working with parents with ID, between 2000 to 2005	**Sex**: 30 females, 19 males **ID level**: Overall: M = 72.8, SD = 8.35 Females: M = 70.02, SD = 6.97, R = 53–80 Males: M = 77.1, SD = 8.72, R = 62–90 **Age** (years): R = 26–58, M = 37.2, SD = 6.98 Females: M = 35.5, SD = 6.19 Males: M = 39.8, SD = 7.5 **Socioeconomic status**: All families low socioeconomic status and receiving statutory income support.	(1) At least one parent with ID or borderline ID (IQ < 75); (2) Impairments of adaptive and/or social functioning, as defined by Wechsler Adult Intelligence Scale‐III and AAMR, respectively	**For mothers only:** **Depression** *N* = 12 (40%) **Anxiety** *N* = 9 (30%) **Mania/hypomania** *N* = 2 (7%) **Obsessive compulsive** *N* = 4 (13%) **Psychosis** *N* = 5 (17%)	Mini psychiatric assessment schedule for adults with a developmental disability	Children's age (years): R = 5–17, M = 9.5 years, SD = 3.4
Tymchuk 1994	United States	Cross‐sectional comparative study	33	Participants of community‐ and university hospital‐based programme designed to provide clinical services to parents with ID	**Sex**: All females **ID level**: Mild (IQ M = 67.8, SD = 6.1) **Marital status**: Married = 52% **Age at birth of first child (years):** M = 21.6 (SD = 4.1) **Education**: Completed high school = 68% **Number of Children**: M = 1.5 **Ethnicity**: Caucasian = 40%, Black or Hispanic = 60% **Income**: Less than $10 000 = 80%	Wechsler Adult Intelligence Scale used to assess IQ	**Depression**: BDI M = 17.58 (SD = 13.98) 13/33 (39%) scored > 17 (depression cutoff)	Becks Depression Inventory	First child age (years): Exposed cohort: M = 3.7, SD = 4.0; Controls: M = 6.7, SD = 4.8
Lindblad et al. 2024	Sweden	Retrospective cohort study (secondary data analysis)	31	Original cohort: 82 children from (1) records at paediatric clinic, (2) records at Board for Provision and Service to the Mentally Retarded, (3) records of special schools for children with ID (4) discussions with principals, school nurses and school psychologists.	**Sex**: 15 females, 16 males **ID level**: Mild **Age at follow‐up (years):** Median = 38, R = 35.5–39.0 **Median age when first child born (years)**: Mothers = 24, IQR 22.5–24.5 Fathers = 27.5, IQR 23.8–30.3 **Median number of children**: Mothers = 3, Fathers = 2	Parents assessed during childhood according to DSM‐IV criteria	**Any psychiatric diagnosis:** *N* = 14 (45%) **Anxiety disorder:** *N* = 8 (26%) **Affective disorder**: *N* = 8 (26%) **ADHD:** *N* = 2 (6%) **Psychotic disorder:** *N* = 1 (3%)	National Patient Register diagnoses‐derived from doctor's visits in specialist care (paediatric or psychiatric care), defined as F0*–F9* according to ICD‐10	Original children's cohort age (years): Median 10, IQR = 5–13, R = 0–21 Ages at follow‐up (years): 0–6: *N* = 31 7–12: *N* = 30 13–21: *N* = 13
Heifetz et al. 2019	Canada	Qualitative study with quantatitive component	12	Urban agency providing parenting support to mothers with ID	**Sex**: All female **ID level**: Mild **Age (years):** M = 35.3, SD = 6.79 **Living situation**: Living alone with child: *N* = 6, living with spouse/partner and child: *N* = 5, living with own mother and child: *N* = 1 **Marital status**: Single: *N* = 7 **Total children**: 28	Previous ID diagnosis (IQ < 70 and below average adaptive skills)	**Depression**: Depressive symptom score M = 13.8 (SD = 5.5) 7/12 (58%) scoring above 13 (clinically significant depressive symptoms)	Glasgow Depression Scale	Children's age (years): M = 11.3, SD = 9.9
Shea et al. 2024	United States	Retrospective cohort (secondary data analysis)	41 854	Data from Centers for Medicare & Medicaid Services for January 2008 to December 2019, including Medicaid Analytic eXtract and Transformed Medicaid Statistical Information System Analytic Files and personal summary and claims files	**Sex**: All female **Age at first delivery (years):** M = 25.5, SD = 6.8 **Age groups (years)**: 14–20 = 28.7%, 21–34 = 61.3%, 35–50 = 9.9% **Ethnicity/race**: White = 44.4%, Black = 34.5%, Hispanic/Latino = 12.5%, Asian/Other Pacific Islander = 1.6%, American Indian/Alaska Native = 1.3%, Multiracial = 0.8%, Missing = 4.8% **Medicaid‐eligibility group**: Poverty = 16.2%, Disability = 68.2%, Other = 15.6%	≥ 1 inpatient or 2 other claims associated with ID diagnosis (ICD‐9‐CM codes 317.xx‐319.xx or ICD‐10‐CM codes F7x)	**ADHD, conduct disorders and hyperkinetic syndrome** *N* = 5498 (13.1%) **Alcohol use disorders** *N* = 1804 (4.3%) **Anxiety disorders** *N* = 10 850 (25.9%) **Bipolar disorder** *N* = 9743 (23.3%) **Depressive disorders** *N* = 13 322 (31.8%) **Drug use disorders** *N* = 5356 (12.8%) **Obsessive–compulsive disorder** *N* = 310 (0.7%) **Personality disorders** *N* = 2259 (5.4%) **Post‐traumatic stress disorder** *N* = 3694 (8.8%) **Schizophrenia** *N* = 4058 (9.7%)	ICD‐9 Diagnosis codes in Medicaid claims	Mental health conditions assessed 1 year before and after delivery
Walton‐Allen 1993	Canada	Cross‐sectional	40	Social service agencies referrals	**Sex**: All female **ID level:** Mild (IQ M = 64.1, R = 49–70) **Age (years)**: M = 32.6, SD = 6.2, R = 19–45 **Marital status**: Single = 52.5% **Previous maternal psychiatric problems:** 15% **Ethnicity**: Asian = 1, Caucasian = 39 **Income**: *N* = 35 (88%) receiving public assistance, *N* = 5 (12%) supported by husband's income; All incomes below 1985 Census Canada low income cutoff ($22 396 for family of 3) **Number of children**: M = 1.7, R = 1–4 **Fathers**: ID = 65%, drug use = 7.5%, alcoholism = 30%	Full scale IQ on Wechsler Adult Intelligence Scale‐Revised: ≤ 70	**Depression**: Asymptomatic (normal) = 23.1% Mild depressive symptoms = 23.1% (score 10–18) Moderate depressive symptoms = 28.2% (score 19–28) Severe depressive symptoms = 25.6% (score 30–63) Clinically significant depressive characteristics: *N* = 25, 62.5% (BDI score ≥ 15)	Becks Depression Inventory	Age of child (years): M = 7.1, SD = 4.24, R = 0.25–13.4
McGaw et al. 2010	United Kingdom	Cross‐sectional (secondary data analysis)	101	Adults involved with Special Parenting Service between 1999 and 2004	**Sex**: 97 female, 4 male **ID level**: Mild (IQ M = 67.6; SD = 5.5; R = 53–74) **Marital status**: couple: *N* = 68; single: *N* = 33 **Total number of children** = 172	Formal assessments and diagnosis using DSM‐IV, AAMR, and ICD‐10 classifications and psychometric testing	‘**Mental health needs**’ High risk group = 29/60 (48%); Low risk group = 14/41 (34%) Total = 43/101 (43%)	Formal diagnoses from case files used as confirmatory evidence to substantiate caseworker reports	Children's ages (years): 0–19
Gaskin and James 2006	United Kingdom	Cross‐sectional comparative study	13 (15 data sets as two women had two pregnancies (took part twice) during study period)	Referrals from health visitors, midwives, community nurses, community consultant paediatricians, social workers and family support workers	**Sex**: All females **ID level:** Mild—borderline (IQ R = 54–73) **Age (years)**: M = 25, R = 18–37 **Ethnicity**: White British: *N* = 11, Indian: *N* = 1, Black British: *N* = 1 **Living situation**: Living with partner: *N* = 7, Separated: *N* = 5, Partner in prison: *N* = 1	IQ < 74, using Wechsler Abbreviated Scale of Intelligence or prior full‐scale IQ assessment	**Postnatal depression**: EPDS M = 10, SD = 6.92; R = 0–21	Edinburgh Postnatal Depression Scale scores and structured interviews with visual scales	All mothers assessed within 12 months postpartum (infant mean age = 5 months)
Tymchuk 1993	United States	Cross‐sectional comparative study	31	Participants of community‐ and university hospital‐based programme designed to provide clinical services to parents with ID	**Sex**: All female **ID level:** Mild (M = 68.4, SD = 6.1) **Marital status**: Married = 52% **Age at birth of first child (years)**: M = 21.7, SD = 4.5 **Education**: Completed high school = 68% **Number of Children**: M = 1.64 **Ethnicity**: Caucasian = 40%, Black or Hispanic = 60% **Income**: Less than $10 000 = 80%	Mothers in special education throughout school years and diagnosed as having ‘mental handicap’	**Schizophrenic disorder:** M = 3.00 (SD = 1.28) **Affective disorder:** M = 3.00 (SD = 1.41) **Psychosexual disorder:** M = 0.83 (SD = 0.80) **Adjustment disorder:** M = 2.86 (SD = 1.48) **Anxiety disorder:** M = 3.06 (SD = 1.41) **Somatoform disorder**: M = 2.86 (SD = 1.52) **Personality disorder:** M = 3.41 (SD = 1.01)	Psychopathology instrument for mentally retarded adults	First child age (years): Exposed cohort: M = 4.4, SD = 5.2; Controls: M = 8.6, SD = 6.2

Abbreviations: AAMR = American Association on Mental Retardation 1992 Manual, DSM‐IV = Diagnostic and Statistical Manual of Mental Disorders, Fourth Edition, ICD‐9/ICD‐10 = International Classification of Diseases, Ninth/Tenth Revision, IQ = intelligence quotient, IQR = interquartile range, M = mean, *N* = number, R = range, SD = standard deviation.

The included studies comprised a total of 42 207 participants (excluding overlapping samples), ranging in age from 18 to 58 years. Of these, 221 participants from seven studies were recruited directly through specialist health, social care and parenting support services for parents with ID. The remaining 41 986 participants were identified from secondary data sources across three studies, including health service and administrative records.

ID had been diagnosed based on DSM‐IV criteria (McGaw et al. 2010; Sterling 1998; Lindblad et al. 2024); ICD‐9 or ICD‐10 criteria (Shea et al. 2024; McGaw et al. 2010); American Association on Mental Retardation 1992 Manual (McGaw et al. 2010, 2007); American Association on Intellectual and Developmental Disabilities Guidance 2011 (Heifetz et al. 2019); and cognitive testing using Wechsler Adult Intelligence Scale‐Revised or Wechsler Abbreviated Scale of Intelligence (Walton‐Allen 1993; Tymchuk 1993, 1994; Gaskin and James 2006). This latter method of diagnosing ID focusing on cognitive testing is in line with ICD‐10 (World Health Organization 1992) and DSM‐III (American Psychiatric Association 1980), which were in use when some of these studies were published. There were no studies identified including participants with genetic syndromes.

Out of the 10 studies, seven included mothers only, whereas three included both mothers and fathers (McGaw et al. 2010; Lindblad et al. 2024; McGaw et al. 2007). In McGaw et al. (2007) it was unclear how many of the fathers had ID. The mean IQ presented for males was 77.1 (SD = 8.72, range = 62–90), whereas female mean IQ was 70.02 (SD = 6.97, range = 53–80). Given that the reported male sample had a mean IQ above the range historically used as an indicator of ID (IQ < 70) (American Psychiatric Association 1994), we considered the female sample more consistent with our inclusion criteria and extracted data from this group only.

The studies included provided sample prevalences for mental disorders overall (McGaw et al. 2010; Lindblad et al. 2024), as well as specific prevalences for depression (Sterling 1998; Tymchuk 1994; Walton‐Allen 1993; Shea et al. 2024; Heifetz et al. 2019; McGaw et al. 2007), anxiety (Shea et al. 2024; Lindblad et al. 2024; McGaw et al. 2007), psychosis/psychotic disorder/schizophrenia (Shea et al. 2024; Lindblad et al. 2024; McGaw et al. 2007), mania/bipolar affective disorder (Shea et al. 2024; McGaw et al. 2007), obsessive–compulsive disorder (OCD) (Shea et al. 2024; McGaw et al. 2007), post‐traumatic stress disorder (PTSD), personality disorders and alcohol/drug use disorders (Shea et al. 2024). Screening tools were used to assess the presence of mental disorders in seven studies. In four studies the screening tools used were originally designed for the general population rather than ID‐specific (Sterling 1998; Tymchuk 1994; Walton‐Allen 1993; Gaskin and James 2006). Three studies used formal diagnoses obtained from service and administrative records (Lindblad et al. 2024; Shea et al. 2024; McGaw et al. 2010).

Of the identified studies, none reported the prevalence of mental disorders specifically during pregnancy. Although mental disorders were assessed over a 2‐year period (1 year before and 1 year after delivery) in Shea et al. (2024), the study did not provide separate prevalence estimates limited to the pregnancy period alone. The only study to administer screening tools to women within 12 months postpartum (children aged 0–1 year) was Gaskin and James (2006). The other eight studies included women with children aged between 0 and 21 years but did not report how many had infants under 1 year, making it unclear how many participants were in the postpartum period.

### 3.2 Risk of Bias

Over half of the studies were assessed as ‘high risk of bias’ (Heifetz et al. 2019; Walton‐Allen 1993; McGaw et al. 2007; Sterling 1998; Tymchuk 1993; Gaskin and James 2006) and four as ‘moderate risk of bias’ (Tymchuk 1994; Lindblad et al. 2024; Shea et al. 2024; McGaw et al. 2010). The main source of bias was in sampling, including restrictive inclusion criteria and low participation rates. A detailed breakdown of MMAT results and justifications for individual study ratings are available in Supporting Information S1: Section S3.

### 3.3 Prevalence of Mental Disorders in Parents With ID

As shown in Table 1, the prevalence in studies that reported ‘mental health needs’ or ‘any psychiatric diagnosis’ in parents with ID was 43% and 45%, respectively (McGaw et al. 2010; Lindblad et al. 2024). Prevalence of depression on health records was 32% (Shea et al. 2024) and up to 63% using screening tools (Walton‐Allen 1993). Anxiety disorder was reported in 26% (Lindblad et al. 2024; Shea et al. 2024) and 30% on screening (McGaw et al. 2007). Psychosis reporting ranged from 3% psychotic disorder (Lindblad et al. 2024) to 17% psychosis symptoms (McGaw et al. 2007). Data for bipolar affective disorder varied from 23% with diagnoses in Medicaid claims (Shea et al. 2024) to 7% screening positive for mania/hypomania (McGaw et al. 2007). Shea et al. (2024) and McGaw et al. (2007) also presented different figures for OCD with 1% clinically diagnosed and 13% screened positive. These differences are likely due to the vastly different study designs, populations included and methods used. Shea et al. (2024), the largest study identified, included data from 41 854 women in the year before and after delivery. This was the only study to report on the numbers of mothers with ID diagnosed with PTSD (9%), personality disorders (5%), alcohol use disorders (4%) and drug use disorders (13%).

### 3.4 Continuous Scores on Screening Tools

Of the seven studies which used screening tools, five of the studies presented average scores obtained on screening tools (Sterling 1998; Heifetz et al. 2019; Tymchuk 1994, 1993; Gaskin and James 2006). In Sterling (1998), the mean score on an adapted version of the Centre for Epidemiologic Studies Depression Scale (CES‐D) was 17.64 (SD 7.68), with the cut‐off score for possible clinical depression of 16. In Heifetz et al. (2019), the mean depressive symptom score on the Glasgow Depression Scale (GDS; developed specifically for those with ID) was 13.8 (SD 5.5), with above 13 indicating clinically significant depressive symptoms. In Tymchuk (1994), mean score on Beck Depression Inventory (BDI) was 17.58 (SD 13.98) with a depression cut‐off score of 17. These studies indicate that a substantial proportion of mothers score above the clinical threshold for possible depression, suggesting elevated levels of depressive symptoms within this population.

Gaskin and James (2006) included a sample of mothers who had given birth in the past 12 months. The mothers completed the Edinburgh Postnatal Depression Scale (EPDS) and structured interviews to assess symptoms of postnatal depression. The mean EPDS score was 10 (SD 6.92; range 0–21), which is a common cut‐off used for depression screening, although studies also use cut‐offs up to 13 (Levis et al. 2020).

Tymchuk (1993) included 31 mothers with ID who completed the self‐report version of the Psychopathology Instrument for Mentally Retarded Adults (PIMRA). This 56‐item tool is organised into eight a priori subscales, with two additional subscales used for mothers with ID. The study reported mean scores on PIMRA subscales rather than specific prevalence percentages of mental disorders. The mothers in the study had the highest mean scores in the subscales for anxiety disorder (M = 3.06, SD = 1.41), schizophrenic disorder (M = 3.00, SD = 1.28) and affective disorder (M = 3.00, SD = 1.41). However, the authors highlight that regarding PIMRA, ‘no decision rules have been developed regarding how many symptoms presented over what periods of time actually mean that a person with ID has a particular disorder’.

### 3.5 Comparative Studies

Four studies compared the prevalence of mental disorders among mothers with ID with that of mothers without ID or non‐parents with ID (Tymchuk 1993, 1994; Lindblad et al. 2024; Shea et al. 2024). These comparative analyses provide important context for interpreting prevalence estimates by examining whether the burden of mental disorders differs according to parental and ID status. A summary of the comparison findings is presented in Table 2.

**TABLE 2:** Comparison studies. Comparisons of rates of mental disorders in mothers with ID with those without ID or non‐parents with ID were provided in four of the studies summarised in the table.

Author, year	ID cohort	ID cohort characteristics	Method to ascertain outcome	Outcome (prevalence)	Comparison group	Comparison group characteristics	Outcome (prevalence) in comparison group	Standardised difference between groups	Factors controlled for in analysis of comparison between groups
Shea et al. 2024	41 854 mothers with ID (no autism) from the Centres for Medicare & Medicaid Services	**Sex**: All female **Age at first delivery (years):** M = 25.5, SD = 6.8 **Age groups (years)**: 14–20 = 28.7%, 21–34 = 61.3%, 35–50 = 9.9% **Ethnicity/race**: White = 44.4% Black = 34.5% Hispanic/Latino = 12.5% Asian/Other Pacific Islander = 1.6% American Indian/Alaska Native = 1.3% Multiracial = 0.8% Missing = 4.8% **Medicaid‐eligibility group**: Poverty = 16.2% Disability = 68.2% Other = 15.6%	ICD‐9 Diagnosis codes in Medicaid claims	**ADHD, conduct disorders and hyperkinetic syndrome:** *N* = 5498 (13.1%) **Alcohol use disorders:** *N* = 1804 (4.3%) **Anxiety disorders:** *N* = 10 850 (25.9%) **Bipolar disorder:** *N* = 9743 (23.3%) **Depressive disorders:** *N* = 13 322 (31.8%) **Drug use disorders:** *N* = 5356 (12.8%) **Obsessive–compulsive disorder:** *N* = 310 (0.7%) **Personality disorders:** *N* = 2259 (5.4%) **Post‐traumatic stress disorder:** *N* = 3694 (8.8%) **Schizophrenia:** *N* = 4058 (9.7%)	438 557 Medicaid‐enrolled mothers without IDD (no autism and no ID)	**Sex**: All female **Age at first delivery (years)**: M = 26.4, SD = 6.3 **Age groups (years):** 14–20 = 21.6% 21–34 = 67.5% 35–50 = 10.9% **Ethnicity/race**: White = 39.4% Black = 22.8% Hispanic = 29.1% Asian/Other Pacific Islander = 3.4% American Indian/Alaska Native = 1.5% Multiracial = 0.4% Missing = 3.4% **Medicaid‐eligibility group**: Disability = 5.9% Poverty = 56.1% Other = 38%	**ADHD, conduct disorders and hyperkinetic syndrome:** *N* = 5498 (1.3%) **Alcohol use disorders:** *N* = 5355 (1.2%) **Anxiety disorders:** *N* = 28 626 (6.5%) **Bipolar diso**rder: *N* = 12 774 (2.9%) **Depressive disorders:** *N* = 32 901 (7.5%) **Drug use disorders:** *N* = 23 581 (5.4%) **Obsessive–compulsive disorder:** *N* = 584 (0.1%) **Personality disorders:** *N* = 1868 (0.4%) **Post‐traumatic stress disorder:** *N* = 5164 (1.2%) **Schizophrenia:** *N* = 1679 (0.4%)	**ADHD, conduct disorders and hyperkinetic syndrome:** aOR = 5.28 (95% CI = 4.99–5.58) **Alcohol use disorders:** aOR = 2.02 (95% CI = 1.87–2.18) **Anxiety disorders:** aOR = 2.49 (95% CI = 2.40–2.58) **Bipolar disorder:** aOR = 3.79 (95% CI = 3.64–3.95) **Depressive disorders:** aOR = 3.01 (95% CI = 2.91–3.10) **Drug use disorders:** aOR = 1.42 (95% CI = 1.36–1.49) **Obsessive–compulsive disorder:** aOR = 2.38 (95% CI = 1.95–2.91) **Personality disorders:** aOR = 5.46 (95% CI = 4.99–5.98) **Post‐traumatic stress disorder:** aOR = 3.38 (95% CI = 3.17–3.60) **Schizophrenia:** aOR = 7.93 (95% CI = 7.30–8.62)	Age at delivery, year of delivery, race/ethnicity and state of residence.
Tymchuk 1993	31 participants of community‐ and university hospital‐based programme designed to provide clinical services to parents with ID	**Sex**: All female **ID level:** Mild (M = 68.4, SD = 6.1) **Marital status**: Married = 52% **Age at birth of first child (years)**: M = 21.7, SD = 4.5 **Education**: Completed high school = 68% **Number of Children**: M = 1.64 **Ethnicity**: Caucasian = 40%, Black or Hispanic = 60% **Income**: Less than $10 000 = 80%	Psychopathology instrument for mentally retarded adults	**Schizophrenic disorder:** M = 3.00 (SD = 1.28) **Affective disorder:** M = 3.00 (SD = 1.41) **Psychosexual disorder:** M = 0.83 (SD = 0.80) **Adjusment disorder:** M = 2.86 (SD = 1.48) **Anxiety disorder:** M = 3.06 (SD = 1.41) **Somatoform disorder:** M = 2.86 (SD = 1.52) **Personality disorder:** M = 3.41 (SD = 1.01)	97 mothers without ID whose children attended daycare in the same areas.	**Sex**: All female **IQ**: Not available **Marital status**: Married = 56% **Age at birth of first child (years)**: M = 22.5, SD = 13.2 **Education**: Completed high school = 55% **Number of children:** M = 2.47 **Ethnicity**: Caucasian = 25%, Black or Hispanic = 75% **Income**: Less than $10 000 = 85%	**Schizophrenic disorder:** M = 1.75 (SD = 1.33) **Affective disorder:** M = 2.18 (SD = 1.58) **Psychosexual disorder:** M = 1.14 (SD = 0.80) **Adjusment disorder:** M = 1.34 (SD = 1.29) **Anxiety disorder:** M = 1.96 (SD = 1.54) **Somatoform disorder:** M = 1.85 (SD = 1.11) **Personality disorder:** M = 2.05 (SD = 1.21)	**Schizophrenic disorder** (*t* = 4.55, *p* = 0.0001) **Affective disorder** (*t* = 2.64, *p* = 0.01) **Psychosexual disorder** (*t* = 1.86, *p* = 0.06) **Adjustment disorder** (*t* = 4.99, *p* = 0.0001) **Anxiety disorders** (*t* = 3.59, *p* = 0.0007) **Somatoform disorders** (*t* = 3.29, *p* = 0.002) **Personality disorders** (*t* = 6.02, *p* = 0.0001)	Not applicable
Tymchuk 1994	33 participants of community‐ and university hospital‐based programme designed to provide clinical services to parents with ID	**Sex**: All females **ID level**: Mild (IQ M = 67.8, SD = 6.1) **Marital status**: Married = 52% **Age at birth of first child (years):** M = 21.6 (SD = 4.1) **Education**: Completed high school = 68% **Number of children**: M = 1.5 **Ethnicity**: Caucasian = 40%, Black or Hispanic = 60% **Income**: Less than $10 000 = 80%	Becks Depression Inventory	**Depression**: BDI M = 17.58 (SD = 13.98) 13/33 (39%) scored > 17 (depression cutoff)	97 mothers without ID, recruited from paediatric clinics, parenting centres and recreation centres.	**Sex**: All female **IQ**: Not available **Marital status**: Married = 56% **Age at birth of first child**: M = 20.4 (SD = 3.5) **Education**: Completed high school = 55% **Number of children**: M = 2.5 **Ethnicity**: Caucasian = 25%, Black or Hispanic = 75% **Income:** Less than $10 000 = 85%	**Depression:** BDI M = 8.31 (SD = 8.19) 13/97 (13%) scored > 17 (depression cutoff)	Mothers with ID scored significantly higher than contrast mothers (*t* = 4.64, 129 dr, *p* < 0.0001)	Whether the mother had problems in addition to ID, history of abuse or neglect as a child, number of children, marital status, housing situation, mother's age at birth of eldest child, whether there was a history of abuse or neglect in the family, whether they were currently receiving aid from a social agency, mother's and eldest child's IQ and eldest child's age.
Lindblad et al. 2024	31 parents from original cohort of 82 children with mild ID	**Sex**: 15 females, 16 males **ID level**: Mild **Education:** Regular = 12 (39%) Special = 19 (61%) **Age at follow‐up (years):** Median = 38, IQR = 35.5–39.0 **Median age when first child born (years)**: Mothers = 24, IQR 22.5–24.5 Fathers = 27.5, IQR 23.8–30.3 **Median number of children**: Mothers = 3, Fathers = 2 **Main source of income:** Regular/subsidised salary = 20 (65%) Sick pension/other social support = 9 (29%) No registered income = 2 (6%) **Criminal convictions:** 14 (45%)	National Patient Register diagnoses	**Any psychiatric diagnosis:** *N* = 14 (45%) **Anxiety disorder:** *N* = 8 (26%) **Affective disorder**: *N* = 8 (26%) **ADHD:** *N* = 2 (6%) **Psychotic disorder:** *N* = 1 (3%)	47 non‐parents from the same original population‐based sample	**Sex**: 16 females, 31 males **ID level**: Mild **Education:** Regular = 9 (19%) Special = 38 (81%) **Age at follow‐up (years):** Median = 36, IQR 34.5–38.0 **Main source of income:** Regular/subsidised salary = 21 (45%) Sick pension/other social support = 24 (51%) No registered income = 2 (4%) **Criminal convictions**: 7 (15%)	**Any psychiatric diagnosis**: *N* = 30 (64%) **Anxiety disorder**: *N* = 13 (28%) **Affective disorder**: *N* = 5 (11%) **ADHD**: *N* = 4 (9%) **Psychotic disorder**: *N* = 4 (9%)	**Psychiatric disorders:** No significant differences between groups (*p* = 0.16)	Not applicable Of note: Education level: No significant differences (*p* = 0.07) Rates of regular salary: Significantly lower in non‐parent (*p* < 0.001) Criminal convictions: Significantly lower in non‐parents (*p* = 0.004)

Abbreviations: aOR = adjusted odds ratio, ASD = autism spectrum disorder, CI = confidence interval, ID = intellectual disability, IDD = intellectual and developmental disabilities, IQ = intelligence quotient, IQR = interquartile range, M = mean, *N* = number, R = range, SD = standard deviation.

Shea et al. (2024) used data from the Centres for Medicare & Medicaid Services (CMS) for January 2008 to December 2019 for 41 854 mothers with an ID diagnosis and a random sample of 438 557 mothers without IDD. Although these results refer to mothers with ‘ID only’, the comparison is against those with ‘no IDD’ as there was not a ‘no ID’ group in the paper. Compared with mothers without IDD, mothers with ID had higher odds for all mental disorders: anxiety disorders 2.49 (95% CI: 2.40–2.58); depressive disorders 3.01 (95% CI: 2.91–3.10); bipolar disorder 3.79 (95% CI: 3.64–3.95); PTSD 3.38 (95% CI: 3.17–3.60); OCD 2.38 (95% CI: 1.95–2.91); schizophrenia 7.93 (95% CI: 7.30–8.62); personality disorder 5.46 (95% CI: 4.99–5.98); alcohol use disorders 2.02 (95% CI: 1.87–2.18); and drug use disorders 1.42 (95% CI: 1.36–1.49).

Tymchuk (1993) compared 31 mothers with a diagnosis of ID from a community and university hospital‐based programme to 97 mothers without ID. Mothers with ID scored consistently higher than mothers without ID. Mean scores on the PIMRA subscales were: schizophrenic disorder 3.00 (SD 1.28) vs. 1.75 (SD 1.33); affective disorder 3.00 (SD 1.41) vs. 2.18 (SD 1.58); adjustment disorder 2.86 (SD 1.48) vs. 1.34 (SD 1.29); anxiety disorder 3.06 (SD 1.41) vs. 1.96 (SD 1.54). Statistical comparisons showed that these differences were significant (Tymchuk 1993). Both Shea et al. (2024) and Tymchuk (1993) indicate that mothers with ID may face elevated risks of mental disorders compared with their peers without ID.

Tymchuk (1994) included the same cohort as Tymchuk (1993) with an additional two mothers. The focus of Tymchuk (1994) however was on the prevalence of depression between mothers with and without ID, using the BDI. This study showed that of 33 mothers with ID, 39% scored above depression cut‐off score of 17, compared with 13% of mothers without ID. In terms of overall scores, mothers with ID scored significantly higher than the non‐ID mothers (M = 17.58, SD = 13.98 vs. M = 8.31, SD = 8.19 respectively; *t* = 4.64, *p* < 0.0001).

In contrast, Lindblad et al. (2024) compared clinical diagnoses of 31 parents (15 mothers and 16 fathers) in a national patient register with clinical diagnoses from 47 non‐parents with mild ID from the same original population‐based sample. The study reported ‘any psychiatric diagnosis’ in *n* = 30 (64%) of non‐parents with ID, compared with *n* = 14 (45%) of parents with ID, although these differences were not significant.

## 4 Discussion

The aim of this review was to synthesise the available evidence on the prevalence of mental disorders among parents with ID. Only 10 studies met inclusion criteria; the majority of studies included parents of children aged 0–21 years, one study focused exclusively on 12 months postpartum and no studies focused specifically during pregnancy. Overall, the studies indicate that 45% of parents with ID are diagnosed with a mental disorder. Depressive disorders are most common, affecting up to 63% of mothers screened and anxiety affecting 30% screened. Where comparison with mothers without ID was available, mothers with ID reported higher rates of mental disorders. However, all the studies were subject to a moderate‐to‐high risk of bias.

### 4.1 Results in Context

The included studies consistently indicate a high burden of mental ill health in parents with ID which aligns with previous research. A review of mental health outcomes across the reproductive life course among women with disabilities also found heightened risks of poor mental health among those with IDD during pregnancy, postpartum and parenting (Deierlein et al. 2024). Although that review employed a broader definition of ID and restricted inclusion to studies with comparison groups, its conclusions are consistent with the present findings. A more focused review of pregnancy outcomes specifically in women with ID demonstrated an increased risk of adverse obstetric outcomes in mothers with ID. Whist this review did not include mental health outcomes, it supports the present findings that parents with ID face disproportionate health disparities (Lo et al. 2025).

Several underlying mechanisms are likely to play a role in the high prevalences of mental disorders observed. Adults with ID experience higher rates of mental disorders than the general population (Santambrogio et al. 2026). Parenthood may introduce additional stressors, including the demands of caring for children, which may be further compounded in parents with ID by socioeconomic disadvantage, reduced social support and increased involvement with statutory services (Booth and Booth 1994; McConnell et al. 2002). Further, barriers to accessing appropriate and timely healthcare may increase the burden of illness (Saeed et al. 2022).

### 4.2 Strengths and Limitations

To our knowledge, this is the first systematic review to synthesise evidence on the prevalence of mental disorders among parents with a specific diagnosis of ID. In doing so, it advances understanding of mental health outcomes in this population, identifies priorities for future research and provides evidence to support improved clinical recognition, service planning and tailored interventions aimed at reducing longstanding mental health inequities.

The reported prevalence estimates should be interpreted with caution because of several limitations of the available evidence. Firstly, definitions of ID varied across studies, reflecting changes in its conceptualisation and diagnostic criteria over time. Secondly, outcomes were assessed inconsistently between studies; most studies used screening tools to identify mental disorders, whereas three relied on formal diagnoses. As diagnostic records capture only recognised cases, they may underestimate prevalence in a population where mental disorders are frequently under‐recognised (NICE 2016). Thirdly, most studies recruited participants directly through specialist services, whereas three used large administrative datasets. Although administrative data provide larger, more representative samples, they rely on the accuracy of diagnostic coding. In contrast, directly recruited samples may provide more detailed clinical information but are smaller and potentially less generalisable. Finally, many studies had small sample sizes and were at high risk of bias.

This review also has limitations. By requiring a formal diagnosis of ID, we may have excluded papers including parents with undiagnosed ID. Despite screening 8303 titles and abstracts, only 10 eligible studies were identified, including two doctoral theses. The small evidence base suggests that the mental health of parents with ID remains a neglected area of research. Due to the paucity of studies and heterogeneity among those studies identified, a meta‐analysis of outcomes was not possible.

### 4.3 Evidence Gaps and Future Research

There is an overall lack of literature examining mental disorders in parents with ID, including those that provide comparisons with populations who are not parents and/or do not have ID. Larger studies are required using population‐based data to ensure a greater representativeness of findings. There is also a preponderance of studies on the common mental disorders of anxiety and depression, with less on more severe mental illness, i.e., psychotic disorders.

In general, knowledge of women's mental health in ID remains limited, with most studies and systematic reviews including predominantly male samples and rarely considering sex‐specific differences (Santambrogio et al. 2026). This review highlights a further evidence gap regarding mental disorders among mothers with ID, particularly during pregnancy and the postpartum period. This lack of evidence is especially concerning given the well‐established importance of maternal mental health during the perinatal period.

This review also indicates a lack of literature pertaining to mental disorders in fathers with ID. Qualitative studies including fathers with ID have highlighted that fathers face mental health challenges which can impact their abilities to parent and support their partners (Symonds et al. 2021). Therefore, studies should include fathers in order for services to learn how to provide them with necessary tailored support.

Finally, all studies identified were from high‐income countries. However, considering that 80% of the global population with disabilities resides in low‐ and middle‐income countries, it is important that the needs of parents with ID in these settings, where healthcare systems and services may be organised differently, are considered in future research (World Health Organization 2011).

## 5 Conclusion

Findings from this review suggest that mothers with ID report disproportionately high rates of mental disorders, although the evidence is limited and at risk of bias. Routine reproductive healthcare encounters present critical opportunities for prevention and intervention, during which all women and their partners should be engaged in discussions about mental health, offered appropriate health advice and provided with individualised, person‐centred care. These opportunities are especially salient for women with ID, who continue to encounter barriers within healthcare systems, including stigma and discrimination, which exacerbate existing health inequities. Further research is warranted to elucidate mental health outcomes during reproductive health periods among women with ID and to inform the development of targeted, evidence‐based interventions.

## Funding

S.R., Doctoral Research Fellow, is funded by the National Institute for Health and Care Research (NIHR) for this research project (NIHR304688). C.A.W. is funded by the NIHR on an Advanced Fellowship (NIHR302950). The views expressed in this publication are those of the author(s) and not necessarily those of the NIHR, UCL, NHS or the UK Department of Health and Social Care.

## Conflicts of Interest

The authors declare no conflicts of interest.

## Supporting information


**Data S1:** Supporting information.

## Data Availability

The data that support the findings of this study are available from the corresponding author upon reasonable request.
